# Interruptions in HIV and Behavioral Health Care for Criminal-Legal Involved People Living with HIV Following Implementation of Decarceration and Shelter in Place in San Francisco, California

**DOI:** 10.1007/s10461-023-04221-x

**Published:** 2023-12-07

**Authors:** A. Asa Clemenzi-Allen, Jillian Hebert, Michael Alistair Reid, Tyler Mains, Hali Hammer, Monica Gandhi, Lisa Pratt, Paul Wesson

**Affiliations:** 1 San Francisco Department of Public Health, San Francisco, CA USA; 2 Division of HIV, Infection Diseases and Global Medicine, University of California, San Francisco, USA; 3 Department of Family and Community Medicine, University of California, San Francisco, USA; 4 Department of Epidemiology and Biostatistics, University of California, San Francisco, USA; 5 798 Brannan St, San Francisco, CA 94103 USA

**Keywords:** Key Populations, Criminal Justice, Care Engagement, Retention in Care, COVID-19 Pandemic

## Abstract

Decarceration policies, enacted for SARS-CoV-2 mitigation in carceral settings, potentially exacerbated barriers to care for people living with HIV (PWH) with criminal legal involvement (CLI) during Shelter-in-Place (SIP) by limiting opportunities for engagement in provisions of HIV and behavioral health care. We compared health care engagement for PWH with CLI in San Francisco, California before and after decarceration and SIP using interrupted time series analyses. Administrative data identified PWH booked at the San Francisco County Jail with at least one clinic encounter from 01/01/2018-03/31/2020 within the municipal health care network. Monthly proportions of HIV, substance use, psychiatric and acute care encounters before (05/01/2019-02/29/2020) and after (03/01/2020-12/31/2020) SIP and decarceration were compared using Generalized Estimating Equation (GEE) log-binomial and logistic regression models, clustering on the patient-level. Of 436 patients, mean age was 43 years (standard-deviation 11); 88% cisgender-male; 39% white, 66% homeless; 67% had trimorbidity by Elixhauser score (medical comorbidity, psychotic disorder or depression, and substance use disorder). Clinical encounters immediately dropped following SIP for HIV (aOR = 0.77; 95% CI: 0.67, 0.90) and substance use visits (aRR = 0.83; 95% CI: 0.70, 0.99) and declined in subsequent months. Differential reductions in clinical encounters were seen among Black/African Americans (aRR = 0.93; 95% CI: 0.88, 0.99) and people experiencing homelessness (aRR = 0.92; 95% CI: 0.87, 0.98). Significant reductions in care were observed for PWH with CLI during the COVID-19 pandemic, particularly among Black/African Americans and people experiencing homelessness. Strategies to End the HIV Epidemic must improve engagement across diverse care settings to improve outcomes for this key population.

**Supplementary Information:**

The online version contains supplementary material available at 10.1007/s10461-023-04221-x.

## Introduction

People living with HIV (PWH) with criminal-legal involvement (CLI) confront substantial individual-level and structural barriers to engagement in care, including substance use, psychiatric disease, and housing instability. Additional challenges to care engagement for this population, posed by how the health care delivery system is structured, includes needing to schedule appointments and maintain insurance coverage [1–5]. Such barriers constitute major challenges to achieving Ending the HIV Epidemic (EtHE) goals in terms of individual outcomes along the HIV care cascade (“Treat” pillar) [6, 7] and mitigating forward transmission of HIV (“Prevent” pillar) [8]. Obstacles to achieving EtHE goals have increased broadly during the COVID-19 pandemic by way of increased subsistence, health, and psychosocial needs through job loss, social isolation and the closure of non-essential medical and behavioral health services [9–12]. However, due to transitioning care to remote/telehealth visits and a shift of the public health workforce to address the COVID-19 response, the capacity of care systems to address these needs has decreased [13, 14]. While incarcerations can disrupt continuity of care for PWH, jail settings can also be leveraged for improving care engagement following release [15]. Widely pursued decarceration policies in response to COVID-19 may have further curtailed opportunities for care engagement for PWH with CLI, above and beyond the well-documented interruptions in community-based HIV care [16–18].

Jails are usually locally resourced and managed and hold individuals awaiting trial, parole, or serving sentences of less than one year [19]. Prisons, on the other hand, are under the jurisdiction of the state or federal government and hold individuals sentenced to more than one year [20]. Although people with CLI carry a disproportionate burden of HIV and social vulnerability, they have been historically understudied, particularly in jail-based settings as opposed to prison. Moreover, longitudinal evaluations that account for incarcerations and community-based HIV and behavioral care can be challenging because of a lack of connectivity between unlinked care databases. Recently, there have been advances in the creation of cross-cutting databases, which can facilitate a granular understanding and ultimate correction of the impact of the COVID-19 pandemic on care engagement [21, 22]. The impact of community and carceral interventions for COVID-19, including decarceration and shelter in place, on healthcare outcomes for PWH with CLI deserves investigation.

On March 17, 2020, San Francisco County (SFC) became the first jurisdiction in the United States to implement a shelter-in-place (SIP) ordinance to mitigate the spread of SARS-CoV-2 [23], which included mandating the delay or shifting of medical visits to telehealth visits. In addition, a strategy of decarceration within the SFC jail reduced the daily jail population by nearly 40% (a reduction of approximately 450 people) between January and April, 2020, a reduction which continued throughout the COVID-19 pandemic [24]. This decarceration policy, which applied to non-violent offenders and patients at high-risk for severe outcomes of COVID-19 disease, was implemented on the basis of inmate age, medical comorbidities, and remaining sentence time (within two months of completing sentence). Moreover, population reductions were maintained through legally mandated alternatives to incarceration, such as attending substance use treatment programs, or releasing patients awaiting trial with non-violent offenses [25]. While this policy may have reduced transmission of SARS-CoV-2 within the jail population [26], it also may have the unintended consequence of interrupting opportunities for engagement in HIV and behavioral health care [27]. Additionally, the communities to which PWH with CLI were released may have limited capacity to maintain care provisions that were administered in the jail system for patients at high risk of disengagement from care [15, 28, 29]. Using an interrupted time series design, we set out to quantify the impact of COVID-19 mitigation policies on HIV, behavioral, and medical care engagement for PWH with CLI in SFC.

## Study Data and Methods

### Setting

The SFC jail (hereafter, “jail”) is the only jail for adults in SFC. The HIV and Integrated Services Ryan White Center of Excellence, a program funded by the Health Resources and Services Administration, provides a patient-centered medical home for any PWH entering this jail system. The program provides HIV, substance use, psychiatric care and case management services to patients to support transitional care planning at the time of entry to and release from jail [15]. Each patient meets with a registered nurse at the time of jail booking, and is encouraged to self-report any chronic medical conditions, including HIV, at that time. The registered nurse then performs a record review to confirm medical history and medication reconciliation to ensure continuation of appropriate medications. Appropriate referrals are made to the jail’s dedicated HIV clinician, HIV nurse case managers and behavioral health specialists, as needed [30]. Consultation by case managers and medical clinicians are required to be completed within 48 h and 7 days, respectively, of jail intake.

### Study Population, Data Collection and Measurements

We constructed a retrospective cohort of PWH who entered the jail at any time between January 1, 2018 and February 29, 2020. Patients were automatically enrolled in HIV care at the time of entry into jail through several mechanisms, including: (1) self-disclosure of diagnosis at the time of medical intake, which is completed by a registered nurse on all people entering jail, (2) review of medical records through the San Francisco Department of Public Health (SFDPH) electronic medical record (EMR), or (3) having a reactive HIV Ag/Ab and confirmatory testing during incarceration. The number of PWH identified through HIV testing during incarceration constitutes a small proportion of the total number of PWH enrolled in care. For instance, 1,668 HIV tests were completed during 2019 and 526 during 2020, which identified nine cases of HIV in 2019 and two cases in 2020. At the time of booking in jail, patients are offered referral to HIV testing by the intake nurse, after which patients are approached and consented for testing by HIV prevention workers (opt-in testing strategy). Carceral stays were abstracted from the Jail Information Management system, the jail-based EMR used during the observation period. Patients were then matched to existing records within the Coordinated Care Management System (CCMS), an administrative data set that integrates data from medical and behavioral health services using electronic health records, social services utilization (e.g., shelter use, housing, jail health services encounters, public entitlements), and mortality from multiple county agencies. CCMS has previously been described elsewhere [21]. Briefly, a CCMS record is created for any patient who is flagged as homeless in any Department of Public Health or county housing system; uses county behavioral health, housing, or jail health services; or uses urgent/emergent medical services across physical health, mental health, substance use, and social health domains. Records of encounters are generated for any service received at a Department of Public Health funded primary care, mental health care, substance use care, housing service and any acute care encounter (e.g., emergency room or hospitalization) in San Francisco County. However, clinical information, such as diagnostic codes, diagnostic evaluations and therapeutic interventions are not included. Historical data are matched at the patient level and continuously updated and integrated into CCMS at the patient level, which holds records for over 450,000 unique adults with up to twenty years of historical social services engagement and diagnostic information.

Outcomes of interest were having an HIV primary care visit, substance use visit, mental health visit, or acute care visit (hospitalization or emergency room visit) during the observation window. Acute care visits were included to serve as a negative control for service encounters (e.g. HIV care encounters, mental health encounters, behavioral health encounters) that were directly impacted by shelter-in-place ordinances. Given that access to in-person HIV and behavioral health services remained intact within jail during the COVID-19 pandemic, a jail custodial stay of at least seven days also counted as an HIV primary care visit, substance use visit, and mental health visit. The first author performed a chart review of a random sample of 10% of all person-incarcerations that confirmed among patients with jail stays of at least seven days, 93% had a visit with a medical clinician, 94% had a visit with a nurse case manager, and 86% had a visit with a social services case manager. Of people with substance use disorder and severe mental illness, here defined as having schizophrena, bipolar disorder or major depressive disorder requiring hospitalization, 92% had a visit with a behavioral health specialist (data not shown). Person-day service encounter data (i.e., date and duration of service encounter) were aggregated up to the person-month level. That is, for each month, a dichotomous variable was created for each outcome to indicate if the individual had accessed that service at any time during that month. Covariates included age, self-reported race and ethnicity, self-reported gender, reported history of homelessness at the time of a service encounter or had accessed homelessness services during the observation period, and having trimorbid disease (e.g. medical co-morbidity, psychotic disorder or depression, and substance use disorder – Appendix A) based on Elixhauser score [31]. Self-reporting of gender, race and ethnicity occurred during the time of CCMS service encounters. In final models, Asian and Pacific Islander categories were combined given small numbers of people who self-reported these racial identities in our cohort. Data for the Elixhauser score is pulled by CCMS from the ICD-9 and ICD-10 codes in the electronic medical record [32]. The ICD codes are used for billing care encounters. CCMS will only have a record of a patient’s diagnosis if these diagnostic codes are also recorded and addressed in the care encounter.

Because this was an open cohort in which people entered at different times and had unequal follow-up, we implemented the following criteria for which to begin counting a patient’s person-time: (1) Patient has a known diagnosis code of HIV using ICD-9/10 codes at any point in the 5-years prior to January 1, 2018, (2) Patient has an episode of incarceration during the pre-intervention period and (3) Patient had an (community-based) HIV visit with a service encounter within three months of the HIV visit during the pre-pandemic period (January 1, 2018 to February 29, 2020). Patients were followed until time of death or end of the cohort observation period (December 31, 2020).

### Analysis Plan

Sociodemographic characteristics were tabulated to describe the characteristics of this cohort.

We implemented an interrupted time series (ITS) analysis, following the framework set by Xiao et al., to measure the impact of COVID-19 mitigation policies (SIP, decarceration) on service engagement for PWH with CLI [33]. ITS is a common quasi-experimental study design and analytic approach to evaluate the longitudinal effects of an intervention introduced at the population level over a clearly defined time period. ITS analysis uses Generalized Estimating Equation (GEE) log-binomial models, clustering on the patient level to account for repeated measures and unequal follow-up. When log-binomial models did not converge, logistic regression models were used instead. ITS models took the following form:

$${Y}_{t}={\beta }_{0}+{\beta }_{1}T+{\beta }_{2}{X}_{t}+{\beta }_{3}\left(T-{T}_{i}\right){X}_{t}+{\beta }_{4}{Z}_{t}$$

Here, β_1_ represents the pre-intervention slope, β_2_ represents the level shift of care engagement at the time of the intervention (March 2020), β_3_ represents the change in slope from the pre-intervention period and β_4_ is the vector of coefficients for the vector of covariates, Z. T represents time (in months) elapsed since the start of the study (May 2019). Subscript *t* is the current time (in month) and subscript *i* is the time (in month) elapsed since the start of the intervention (March 2020). Finally, Y is a dichotomized outcome indicating whether the patient had a service encounter at month *t*. We ran these models separately for each service encounter outcome (HIV care visit, substance use, mental health, acute care).

We restricted the observation window for the ITS analysis to May 2019 to December 2020 in order to minimize the potential for observation bias given that people with care encounters at the beginning of cohort enrollment (i.e., January 2018 – April 2019) may have higher rates of care engagement. These higher rates of care engagement were spurious and an artifact of constructing the analytic cohort; as part of the criteria to begin observation, patients needed a service encounter within three months of an HIV diagnosis or visit. Adjusted models controlled for age, sex, gender, race and ethnicity, trimorbidity, and history of homelessness.

To disaggregate care interruptions that resulted from decarceration and SIP individually, we repeated unadjusted and adjusted analyses that restricted to prolonged jail stays (e.g. ≥7 days) and community-based HIV care visits, respectively, and compared rates of care encounters prior to, immediately following and in the months subsequent to the implementation of decarceration and SIP.

Additionally, we performed stratified analyses to examine evidence for effect measure modification. Models were stratified, separately, by race and ethnicity, gender, and history of homelessness. These stratifying variables were determined a priori under the hypothesis that groups from socially marginalized backgrounds would have differential care engagement experiences during the COVID-19 pandemic. Patients were censored at the time of death or six-months following their last care encounter during the observation period.

### Ethics Statement

This study was reviewed and approved by the UCSF IRB (IRB# 20-31387).

## Study Results

### Cohort Characteristics

We identified 436 people incarcerated from January 1, 2018 – February 29, 2020 who met our inclusion criteria. Of these people, the majority identified as cis-gender male (n = 382, 88%) and 137 (31%) were over the age of 50. A plurality of the analytic cohort identified as White (n = 169, 39%), followed by 154 (35%) who identified as Black/African-American. Nearly half of the cohort (47%) had trimorbidity, according to the Elixhauser score (medical comorbidity, substance use comorbidity, and psychotic disorder or depression), and 288 (66%) experienced homelessness at some point during the observation period (Table 1).

**Table 1:** Sociodemographic characteristics of analytic cohort of people living with HIV with criminal legal involvement in San Francisco, CA, January 2018 through April 2019 (N = 436)

Sociodemographic variable	N (%)
**Age**	
<30	45 (10)
30–39	149 (34)
40–49	105 (24)
50–59	105 (24)
60+	32 (7.3)
**Gender**	
Male	382 (88)
Female	45 (10)
Transgender	9 (2.1)
**Race/Ethnicity**	
White	169 (39)
Black/African American	154 (35)
Latinx	74 (17)
API	14 (3.2)
Other	25 (5.7)
**History of Homelessness**	288 (66)
**Elixhauser score**	
**< 3**	229 (52.7)
**3 (Trimorbidity)**	207 (47)

Transgender = male to female transgender, female to male transgender, gender non-binary or self-reported ‘other’. API = Asian and Pacific Islander; “Other” Race/Ethnicity category includes Indigenous American, self-reported ‘other’. Elixhauser score determined based on summation of diagnosed comorbidity categories (medical, substance use, mental health).

### Interrupted Time Series Results

**Fig. 1:**
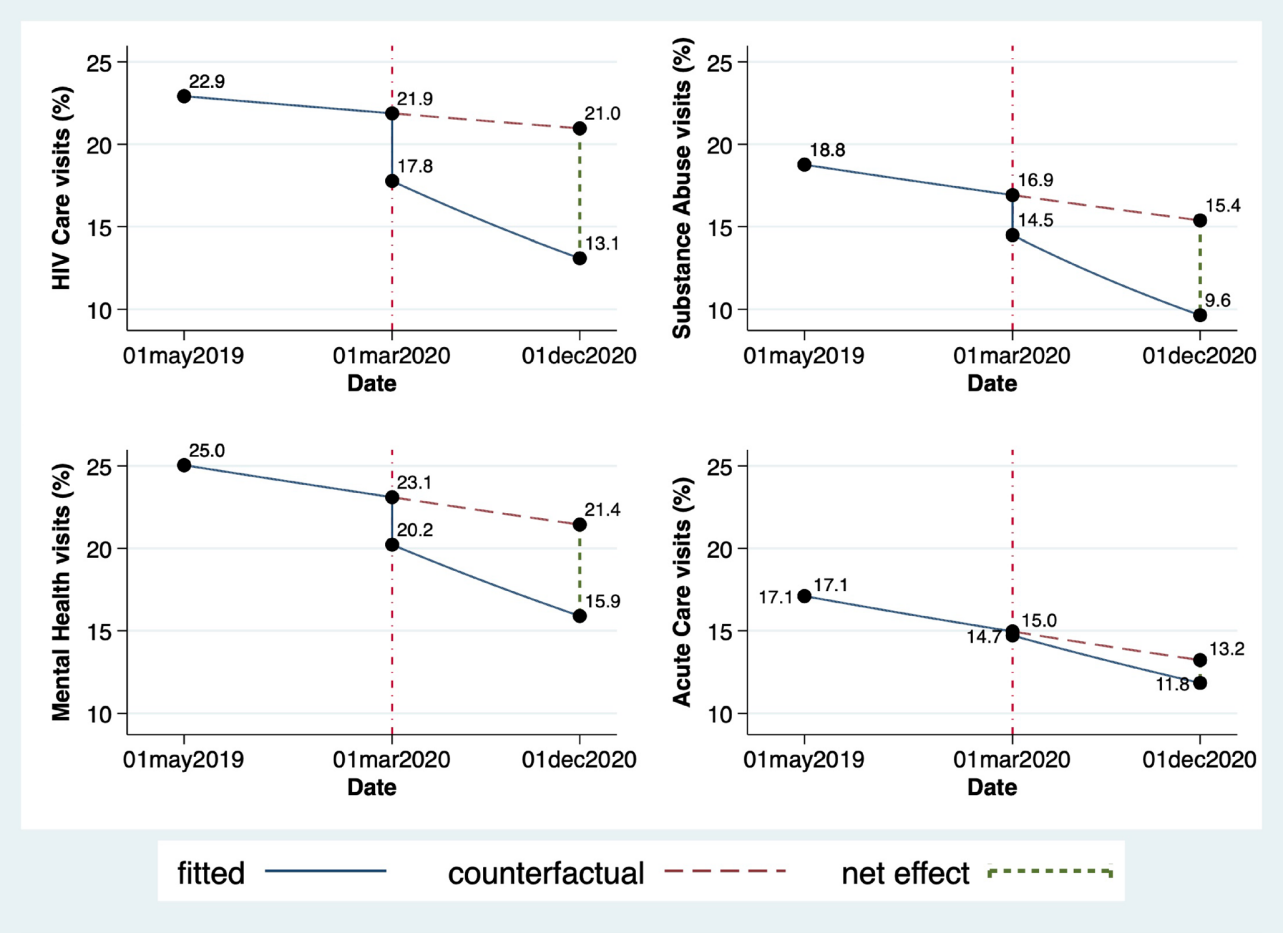
Modeled monthly health care utilization and interruption for People Living with HIV with Criminal Justice Involvement in San Francisco, May 2019 through December 2020 Fitted = modeled proportion of cohort utilizing health service based on observed cohort data. Counterfactual = expected proportion of cohort utilizing health service in the absence of March 2020 decarceration and COVID-19 mitigation policies. Net effect = difference between fitted and counterfactual proportion of cohort utilizing health service at endline (December 2020)

The pre-intervention slope was null in all four domains of health care encounters (HIV visit, Substance Use, Mental Health, Acute care), indicating no change in the probability of care engagement during the pre-pandemic period (May 2019 to February 2020). In separate unadjusted ITS models, we observed substantial and significant reductions in HIV care visits (RR 0.77, p-value 0.001, 95% CI 0.67–0.90) and substance use (RR 0.83, p-value 0.033, 95% CI 0.70–0.99), but not in mental health visits (RR 0.89, p-value 0.083, 95% CI 0.77–1.02) or acute care visits (RR 0.97, p-value 0.70, 95% CI 0.83–1.13) at the time of decarceration and SIP (March, 2020). Moreover, we observed persistent reductions in the risk of engagement in HIV care (RR 0.96, p-value 0.004, 95% CI 0.93–0.99), substance use visits (RR 0.95, p-value 0.006, 95% CI 0.91–0.99), mental health (RR 0.97, p-value 0.039, 95% CI 0.95–1.00) and acute care visits (RR 0.97, p-value 0.028, 95% CI 0.94–1.00) in the subsequent months following decarceration and SIP (Table 2; Fig. 1). These trends persisted in adjusted models without significant attenuation (Table 3).

**Table 2:** Unadjusted interrupted time series analysis, by health service encounter type, for people living with HIV with criminal legal involvement in San Francisco, CA, May 2019 through December 2020

	HIV Visits		Mental Health Encounters		Substance Use Encounters		Acute Care Visits	
	RR (95% CI)	p-value	RR (95% CI)	p-value	RR (95% CI)	p-value	RR (95% CI)	p-value
Pre-SIP	1 (0.98, 1.01)	0.37	0.99 (0.98, 1.01)	0.37	0.99 (0.97, 1.01)	0.285	1 (0.99, 1.02)	0.80
SIP	**0.77 (0.66, 0.90)**	**0.001**	0.89 (0.77, 1.02)	0.083	**0.83 (0.70, 0.99)**	**0.033**	0.97 (0.83, 1.13)	0.70
SIP*Time	**0.96 (0.93, 0.99)**	**0.024**	0.98 (0.95, 1.01)	0.259	0.96 (0.92, 1.00)	0.058	**0.97 (0.94, 1.00)**	**0.045**
Post-SIP	**0.96 (0.93, 0.96)**	**0.004**	**0.97 (0.95, 1.00)**	**0.039**	**0.95 (0.91, 0.99)**	**0.006**	**0.97 (0.94, 1.00)**	**0.028**

RR = relative risk. CI = Confidence Interval. SIP = Shelter in Place. Pre-SIP = RR of having clinical encounter by month during the pre-Shelter in Place observation period. SIP = RR of having clinical encounter at the time of SIP (e.g., March 2020). SIP*Time = interaction term between SIP and time (months). Significant RR indicates a statistically different slope from the pre-SIP period. Post-SIP = monthly RR of having clinical encounter following SIP

**Table 3:** Adjusted interrupted time series analysis, by health service encounter type, for people living with HIV with criminal-legal involvement in San Francisco, CA, May 2019 through December 2020

	HIV Visits		Mental Health Encounters		Substance Use Encounters		Acute Care Visits	
	RR (95% CI)	p-value	OR (95% CI)	p-value	RR (95% CI)	p-value	OR (95% CI)	p-value
Time								
Pre-SIP	0.99 (0.98, 1.01)	0.333	0.99 (0.97, 1.01)	0.294	0.99 (0.97, 1.01)	0.191	0.99 (0.97, 1.01)	0.294
SIP	**0.77 (0.67, 0.90)**	**0.001**	0.85 (0.71, 1.02)	0.078	**0.83 (0.70, 0.99)**	**0.034**	0.85 (0.71, 1.02)	0.977
SIP*Time	**0.97 (0.94, 1.00)**	**0.043**	0.98 (0.94, 1.02)	0.361	0.96 (0.92, 1.01)	0.084	0.98 (0.94, 1.02)	0.361
Post-SIP	**0.96 (0.93, 0.99)**	**0.007**	**0.97 (0.94, 1.00)**	**0.045**	**0.95 (0.91, 0.99)**	**0.007**	0.97 (0.94, 1.01)	0.13
Gender								
Male	REF	REF	REF	REF	REF	REF	REF	REF
Female	0.9 (0.68, 1.19)	0.473	**1.82 (1.04, 3.21)**	**0.037**	1.25 (0.79, 1.99)	0.348	**1.53 (1.04, 2.25)**	**0.032**
Transgender	0.99 (0.68, 1.44)	0.991	1.38 (0.42, 4.60)	0.597	0.26 (0.06, 1.03)	0.056	1.36 (0.57, 3.25)	0.539
Race/Ethnicity								
White	REF	REF	REF	REF	REF	REF	REF	REF
Black	1.17 (0.95, 1.43)	0.143	1.31 (0.86, 2.00)	0.205	1.28 (0.87, 1.87)	0.208	0.96 (0.71, 1.30)	0.79
Latinx	1.21 (0.95, 1.54)	0.125	1.3 (0.77, 2.19)	0.319	1.03 (0.63, 1.70)	0.899	1.04 (0.70, 1.54)	0.857
API	0.62 (0.33, 1.17)	0.14	0.98 (0.31, 3.07)	0.979	0.94 (0.36, 2.45)	0.899	1.33 (0.80, 2.22)	0.273
Other	1.07 (0.73, 1.57)	0.732	1.02 (0.50, 2.08)	0.958	0.9 (0.44, 1.82)	0.768	0.89 (0.52, 1.52)	0.659
Age								
<30	REF	REF	REF	REF	REF	REF	REF	REF
30–39	**1.46 (1.05, 2.01)**	**0.023**	1.74 (0.92, 3.30)	0.089	2.09 (0.98, 4.47)	0.056	0.87 (0.55, 1.38)	0.555
40–49	**1.49 (1.06, 2.07)**	**0.023**	1.33 (0.67, 2.63)	0.42	2.16 (0.99, 4.75)	0.054	1.24 (0.76, 2.02)	0.5
50–59	**1.48 (1.05, 2.07)**	**0.023**	0.96 (0.48, 1.90)	0.896	1.72 (0.78, 3.78)	0.176	0.76 (0.47, 1.24)	0.238
60+	1.27 (0.79, 2.07)	0.326	1 (0.39, 2.59)	0.958	1.72 (0.68, 4.37)	0.251	1.33 (0.68, 2.63)	0.403
Trimorbid	0.91 (0.77, 1.07)	0.222	1.09 (0.78, 1.50)	0.622	1.06 (0.80, 1.41)	0.693	1.12 (0.87, 1.46)	0.53
Homeless	**1.58 (1.41, 1.78)**	**< 0.001**	**1.24 (1.05, 1.48)**	**0.013**	**1.2 (1.03, 1.40)**	**0.017**	**5.82 (4.73, 7.15)**	**< 0.005**

Models adjust for gender (cis-male, cis-female), age by decade, Trimorbidity (e.g. having chronic medical, substance use and psychotic disorder or depression based on Elixhauser score), homeless (e.g. having reported history of homelessness at any time during the observation period). REF = Reference Level. RR = Relative Risk. OR = Odds Ratio. CI – Confidence Interval. SIP = Shelter in Place. Pre-SIP = RR of having clinical encounter by moth during pre-Shelter in Place observational period. SIP = RR of having clinical encounter at the time of SIP (e.g. March 2020). SIP*Time = interaction term between SIP and time (months). Significant RR indicates a statistically significant different slope from the pre-SIP period. Post-SIP = monthly RR of having clinical encounter following SIP

### Sensitivity Analysis

In an unadjusted analysis, restricting to community-based HIV care showed a substantial and statistically significant reduction in HIV visits in the month immediately following SIP and decarceration (RR 0.77, p-value 0.11, 95% CI 0.63–0.94) and a trend towards reduced care visits in the months subsequent to SIP and decarceration (RR 0.96, p-value 0.052, 95% CI 0.94–1.01). Results did not substantially change with full adjustment. A separate unadjusted analysis restricting to prolonged jail stays (i.e. ≥7 days) showed a trend towards a reduction in prolonged jail stays immediately following SIP and decarceration (RR 0.77, p-value 0.79, 95% CI 0.58–1.03) and a substantial and statistically significant reduction in prolonged jail stays in the months subsequent to SIP and decarceration (RR 0.93, p-value 0.20, 95% CI 0.87–0.99). Results did not substantially change in fully adjusted models (see Appendix Tables 1 and 2).

### Trends Among Key Populations (Table 4)

**Table 4:** Care Engagement by Month Before, Immediately After and Over Time Following Shelter in Place within Key Populations

	Before Shelter in Place	Immediately After Shelter in Place	Over Time Following Shelter in Place
	aRR (95% CI)	aRR (95% CI)	aRR (95% CI)
**HIV Engagemen**t			
Cis-Male	0.99 (0.97, 1.01)	0.68 (0.55, 0.85)**	0.95 (0.92, 0.99)*
Cis-Female	0.97 (0.92, 1.03)	0.58 (0.34, 1.00)*	0.98 (0.88, 1.08)
White	1.00 (0.97, 1.02)	0.63 (0.45, 0.88)**	0.98 (0.98, 1.05)
Black	1.00 (0.97, 1.03)	0.68 (0.49, 0.95)*	0.93 (0.88, 0.99)*
Latino/a	0.98 (0.97, 1.03)	0.71 (0.39, 1.28)	0.94 (088, 1.01)
Homelessness	1.03 (0.99, 1.06)	0.65 (0.46, 0.91)**	0.92 (0.87, 0.98)*
No Homelessness	0.98 (0.96, 1.00)*	0.70 (0.54, 0.90)**	0.96 (0.92, 1.00)
**Substance Use Engagement**			
Cis-Male	0.99 (0.96, 1.01)	0.74 (0.58, 0.94)*	0.95 (0.90, 1.00)*
Cis-Female	0.97 (0.90, 1.05)	0.89 (0.54, 1.46)	0.88 (0.77, 1.00)*
White	0.99 (0.97, 1.03)	0.70 (0.50, 0.97)*	0.99 (0.92, 1.07)
Black	1.00 (0.97, 1.03)	0.64 (0.46, 0.90)**	0.92 (0.88, 1.00)*
Latino/a	0.96 (0.89, 1.03)	1.29 (0.70, 2.38)	0.88 (0.79, 0.99)*
Homelessness	1.01 (0.97, 1.04)	0.69 (0.50, 0.95)*	0.94 (0.86, 1.02)
No Homelessness	0.98 (0.97, 1.01)	0.76 (0.60, 0.96)*	0.97 (0.93, 1.00)*
**Mental Health Engagement**			
Cis-Male	0.99 (0.97, 1.01)	0.88 (0.75, 1.03)	0.99 (0.95, 1.02)
Cis-Female	1.00 (0.96, 1.04)	0.84 (0.66, 1.06)	0.97 (0.88, 1.05)
White	0.99 (0.97, 1.02)	0.88 (0.69, 1.12)	0.99 (0.94, 1.05)
Black	0.98 (0.96, 1.01)	0.75 (0.60, 0.94)*	0.99 (0.94, 1.05)
Latino/a	1.03 (0.99, 1.07)	0.97 (0.75, 1.25)	0.92 (0.86, 0.99)*
Homelessness	1.01 (0.99, 1.03)	0.89 (0.72, 1.09)	0.97 (0.93, 1.02)
No Homelessness	0.99 (0.97, 1.01)	0.85 (0.72, 1.00)*	0.99 (0.95, 1.03)
**Acute Care Engagement**			
Cis-Male	1.00 (0.98, 1.01)	1.06 (0.89, 1.25)	0.97 (0.93, 1.00)*
Cis-Female	1.02 (0.98, 1.06)	0.66 (0.47, 0.94)*	0.97 (0.88, 1.07)
White	0.98 (0.98, 1.03)	1.38 (1.02, 1.86)*	0.95 (0.90, 1.00)*
Black	0.98 (0.94, 1.01)	0.85 (0.60, 1.21)	0.97 (0.91, 1.04)
Latino/a	1.01 (0.96, 1.06)	0.66 (0.39, 1.13)	1.00 (0.91, 1.10)
Homelessness	0.98 (0.95, 1.01)	1.24 (0.90, 1.70)	0.92 (0.87, 0.98)**
No Homelessness	0.99 (0.97, 1.01)	0.84 (0.66, 1.05)	1.01 (0.97, 1.06)

aRR = adjusted relative risk. CI = Confidence Interval. *signifies p-value < 0.05; **signifies p-value < 0.005. The following categories were not included in these models due to small sample size: Transgender, Asian/Pacific Islander race/ethnicity and Other race/ethnicity. Homelessness = having reported or documented history of homelessness at any time during the observation period

Stratified models revealed similar trends for care engagement immediately following SIP and decarceration, but differed in care engagement in the months following the interventions within the pre-identified groups. Note that for the following effect modification analyses results compare pandemic care engagement to pre-pandemic care engagement within strata of specified groups.

#### Race and Ethnicity

Both White and Black/African American patients experienced an immediate and significant reduction in HIV care visits following SIP; however, the continued reduction in HIV care visits observed in the main analysis was only seen among Black/African American patients (aOR = 0.93, 95% CI: 0.88–0.99). There was no significant reduction in HIV care visits for Latinx patients. For substance use visits, only Black/African American patients experienced an immediate and significant decline in care engagement at the time of SIP (aOR = 0.72, 95% CI: 0.55–0.94). Both Black/African American (aOR = 0.92, 95% CI: 0.86-1.00) and Latinx patients (aOR = 0.88, 95% CI: 0.79–0.99) experienced a continued reduction in substance use visits in the months following SIP.

#### Gender

Both cismale and cisfemale patients experienced an immediate and significant drop in HIV care visits at the time of SIP, but only cismale patients had a sustained reduction in care visits in the months following SIP (aOR = 0.95; 95% CI: 0.92–0.99). In contrast, for substance use visits, both cismale and cisfemale patients had a significant reduction in care visits in the months following SIP, but only cismale patients had an immediate reduction in substance use visits at the time of SIP (aOR = 0.74; 95% CI: 0.58–0.94). There were no significant differences for accessing acute care services across genders.

#### Housing Status

A history of homelessness was associated with persistent reductions in HIV care visits (aOR = 0.92; 95% CI: 0.87–0.98) and in acute care visits (aOR = 0.92; 95% CI: 0.87–0.98) in the months following SIP.

## Discussion

In this ITS analysis, we investigated the impacts of COVID-19 mitigation policies (SIP, decarceration) on engagement in care for PWH with CLI in San Francisco by leveraging a large and unique county-level administrative health care dataset. While our results demonstrate substantial and significant reductions in care engagement during the COVID-19 pandemic for HIV care and substance use encounters, we did not detect similar changes in care engagement for mental health visits or acute care visit. The general trend of reductions in HIV care engagement for this cohort following SIP and decarceration is especially concerning given its implications for initiatives such as Ending the HIV Epidemic [34]. Disengagement from HIV care likely signals interruptions in antiretroviral medication adherence, which could lead to viral rebound and forward community HIV transmission.

Moreover, our analysis showed evidence for effect modification, revealing unequal disengagement from care during the COVID-19 pandemic for Black/African Americans and Latinx patients. This is especially alarming because, prior to the pandemic, municipalities that reported overall success in meeting targets to control the local HIV epidemic also reported that these targets were not met for their Black/African American and Latinx populations [35, 36]. Our findings suggest that these pre-existing disparities have likely worsened due to differential disengagement from care, at least among PWH with CLI. Additional resources are needed to mitigate the cumulative impact of community-level COVID-19 mitigation efforts. Reversing these trends will require interventions tailored to specific patient populations while also addressing the syndemics of substance use, mental health, homelessness and incarceration in racial and ethnic minorities as well as other intersectional challenges that serve as barriers to equitable access to health care [37, 38].

Lastly, our research demonstrates the benefits of using large, county-level datasets to identify granular care engagement patterns above and beyond HIV care alone. Additional benefits compared to clinical cohorts include the ability to capture service engagement across myriad service types (e.g. substance use, psychiatric, acute care and criminal-legal involvement), identifying PWH who are disengaged from community-based primary care and having up-to-date death index data. While the up-front investment needed to establish new partnerships, such as identifying champions, adhering to data privacy and bureaucratic procedures, can be onerous, nurturing these relationships provides an opportunity to leverage the funding, research and dissemination capacity of academic researchers to produce targeted, actionable research that responds to public health problems. The underappreciated work in developing and sustaining public health-academic partnerships creates an infrastructure for data monitoring and surveillance, implementation and adaptation of programs for HIV treatment and prevention [39]. The successful collaboration that we nurtured throughout this research will be leveraged to inform real-world programmatic changes to address the care gaps that we identified and to continue research that refines and deepens comprehensive care engagement for PWLH with criminal-legal involvement.

Limitations in our study may have arisen from several factors relating to our cohort design and statistical analysis. First, the use of administrative data may not reflect all care engagement patterns given the potential for use of venues not included in the county health system database for medical, substance use or mental health care (e.g. private clinics, community-based organizations, out of jurisdiction care) [40]. To limit this potential bias of misclassifying people as not engaged in care who may be engaged in care outside of SFC, we limited our cohort to patients who had a history of community-based HIV care engagement. Second, because the outcome of interest included HIV care rendered in jail and in community-based HIV care settings, we were unable to directly disaggregate the impact of reductions in care encounters due to decarceration and reductions in community-based HIV care individually. However, our sensitivity analyses suggest that the immediate reductions in overall HIV care engagement may have been largely attributed to reductions in community-based care engagement at the time of SIP, whereas the continued decline in HIV care engagement following SIP may have been attributed more to reductions in prolonged jail stays. Relatedly, due to the limitations of our administrative data set, we did not have clear documentation of HIV care visits received while in jail. Due to the high correlation of HIV care visits and prolonged jail stays from a chart review of a subset of patients (see Methods), we equated jail stays of at least seven days with HIV care visits for the purpose of this analysis. The direction of the bias from this potential outcome misclassification is unclear as patients in jail for fewer than seven days may have had an HIV care visit, just as patients in jail for greater than seven days could have not had an HIV care visit. However, our sensitivity analysis, where we conservatively classified the outcome as community-based HIV care visits only, suggests a slight attenuation of results. Furthermore, we evaluated relatively short-term outcomes in our ITS analysis, with our post-SIP observation window covering only nine months. Therefore, our results speak to the immediate systemic disruption of COVID-19 and mitigation policies. Extending the follow-up period will be necessary to determine if the patterns of health care disengagement persisted, or if the trend eventually reversed as the health care system adapted to the evolving pandemic. Future research should also include viral load measurements, which were absent from our administrative data set, as a more informative metric of successful HIV care engagement. Lastly, San Francisco County is unique as an environment given its long history of public health [41] and academic research collaborations, including the development of national models in HIV care, our methods and findings may not be generalizable [42, 43]. More research is needed to disaggregate the changes in HIV care engagement from unique changes in HIV care rendered within each care setting, particularly as public health measures in response to the COVID-19 pandemic have evolved since December 2020.

## Conclusions

In conclusion, we observed immediate reductions in HIV, substance use, and mental health care engagement for PWH with CLI following implementation of SIP and decarceration with persistent reductions in care during 2020. Furthermore, differential reductions in care engagement for Black/African Americans and people experiencing homelessness suggest disproportionate impacts related to structural racism and high social vulnerability. Strategies to improve these gaps in care are urgently needed to meet domestic HIV care outcomes goals, particularly among vulnerable populations [34], which can be informed by use of large-scale, integrated data systems to identify gaps in care access. Moreover, structural changes implemented during the COVID-19 pandemic to support long-term care engagement, such as easing enrollment restrictions for Ryan White HIV/AIDS Program, providing housing services, and addressing other unmet needs must continue [14]. Moreover, the rapid expansion and increased capacity of the public health workforce - the backbone of the COVID-19 response - can serve as a foundation to address these major gaps in HIV care if policy-makers can realign disease control priorities and support sustainability in an overburdened work-force [14]. Finally, our study provides a cautionary note on the potential unintended consequences of interventions aimed to protect people. Several studies have demonstrated that decarceration can effectively reduce COVID-19 transmission [44] and, in fact, it is mass incarceration that is associated with increased COVID-19 transmission among incarcerated individuals, carceral staff, and the surrounding community [45]. However, given the role that carceral settings play in providing health care to populations that disproportionately face intersecting and compounding social vulnerabilities (e.g., unstable housing, low income, substance use, mental illness), such decarceration polies must also be paired with interventions to re-engage this population in community health care settings. This is especially critical for PWH as lapses in HIV care visits can result in antiretroviral medication nonadherence and uncontrolled viral load, ultimately compromising personal health and increasing the risk of community spread.

### Electronic Supplementary Material

Below is the link to the electronic supplementary material.

Supplementary Material 1

## Declarations

### Disclamer

Findings have been accepted for poster presentation at AIDS 2022, Montreal, Canada (July 29th – August 2nd, 2022).
