# Supplementary material for: Interruptions in HIV and Behavioral Health Care for Criminal-Legal Involved People Living with HIV Following Implementation of Decarceration and Shelter in Place in San Francisco, California
Source: AIDS Behav. 2023 Dec 7;28(3):1093–103. doi: 10.1007/s10461-023-04221-x (PMC10896806; doi:10.1007/s10461-023-04221-x)
Supplement: Supplementary file 1 — Supplementary Material 1 [file 10461_2023_4221_MOESM1_ESM.docx]

Appendix Tables

Appendix A. List of Medical, Mental Health and Substance Use Disorder Elixhauser Comorbidities.

| **Elixhauser Comorbidities** | |
| --- | --- |
| **Medical** | AIDS/HIV, blood loss anemia, cardiac arrhythmias, chronic pulmonary disease, coagulopathy, congestive heart failure, deficiency anemia, complicated diabetes, uncomplicated diabetes, fluid and electrolyte disorders, complicated hypertension, uncomplicated hypertension, hypothyroidism, liver disease, lymphoma, metastatic cancer, obesity, other neurological disorders, paralysis, peptic ulcer disease excluding bleeding, peripheral vascular disease, pulmonary circulation disorder, renal failure, rheumatic arthritis and collagen vascular disease, solid tumor without metastasis, valvular disease, weight loss |
| **Mental Health** | Depression and psychoses |
| **Substance Use Disorder** | Alcohol use disorder and drug use disorder |

**Table 1.** Unadjusted and adjusted interrupted time series analysis of community-based HIV care visits for people living with HIV with criminal-legal involvement in San Francisco, CA, May 2019 through December 2020.

|  | Unadjusted | | Adjusted | |
| --- | --- | --- | --- | --- |
|  | **RR (95% CI)** | **p-value** | **RR (95% CI)** | **p-value** |
| **Time** |  |  |  |  |
| **Pre-SIP** | 1.01 (1.00, 1.03) | 0.145 | 1.01 (0.99, 1.02) | 0.459 |
| **SIP** | **0.77 (0.63, 0.94)** | **0.011** | **0.78 (0.64, 0.95)** | **0.013** |
| **SIP*Time** | 0.96 (0.92, 1.00) | 0.052 | 0.97 (0.93, 1.01) | 0.179 |
| **Post-SIP** | 0.97 (0.94, 1.01) | 0.141 | 0.98 (0.95, 1.02) | 0.254 |
| **Gender** |  |  |  |  |
| **Male** |  |  | REF | REF |
| **Female** |  |  | 0.95 (0.69, 1.32) | 0.77 |
| **Transgender** |  |  | 1.08 (0.61, 1.91) | 0.794 |
| **Race/Ethnicity** |  |  |  |  |
| **White** |  |  | REF | REF |
| **Black** |  |  | 0.96 (0.73, 1.25) | 0.751 |
| **Latinx** |  |  | 1.27 (0.93, 1.73) | 0.127 |
| **API** |  |  | 0.62 (0.29, 1.36) | 0.233 |
| **Other** |  |  | 0.99 (0.63, 1.54) | 0.949 |
| **Age** |  |  |  |  |
| **<30** |  |  | REF | REF |
| **30-39** |  |  | 1.07 (0.71, 1.61) | 0.761 |
| **40-49** |  |  | 1.26 (0.82, 1.93) | 0.287 |
| **50-59** |  |  | **1.56 (1.03, 2.35)** | **0.035** |
| **60+** |  |  | 1.30 (0.74, 2.28) | 0.359 |
| **Trimorbid** |  |  | 0.95 (0.77, 1.17) | 0.629 |
| **Homeless** |  |  | **2.35 (2.02, 2.74)** | **<0.005** |

Models adjust for gender (cis-male, cis-female), age by decade, Trimorbidity (e.g having chronic medical, substance use and psychiatric disease based on elixhauser score), homeless (e.g. having reported history of homelessness at any time during the observation period). REF = Reference Level. RR = Relative Risk. OR = Odds Ratio. CI – Confidence Interval . SIP = Shelter in Place. Pre-SIP = RR of having clinical encounter by moth during pre-Shelter in Place observational period. SIP = RR of having clinical encounter at the time of SIP (e.g. March 2020). SIP*Time = interaction term between SIP and time (months). Significant RR indicates a statistically significant different slope from the pre-SIP period. Post-SIP = monthly RR of having clinical encounter following SIP.

**Table 2.** Unadjusted and adjusted interrupted time series analysis of prolonged jail stays (≥7 days) for people living with HIV with criminal-legal involvement in San Francisco, CA, May 2019 through December 2020.

|  | Unadjusted | | Adjusted | |
| --- | --- | --- | --- | --- |
|  | **RR (95% CI)** | **p-value** | **RR (95% CI)** | **p-value** |
| **Time** |  |  |  |  |
| **Pre-SIP** | **0.97 (0.95, 1.00)** | **0.047** | 0.98 (0.95, 1.00) | 0.053 |
| **SIP** | 0.77 (0.58, 1.03) | 0.079 | 0.78 (0.59, 1.03) | 0.082 |
| **SIP*Time** | 0.95 (0.89, 1.02) | 0.180 | 0.95 (0.89, 1.02) | 0.173 |
| **Post-SIP** | **0.93 (0.87, 0.99)** | **0.021** | **0.93 (0.87, 0.99)** | **0.019** |
| **Gender** |  |  |  |  |
| **Male** |  |  | REF | REF |
| **Female** |  |  | 1.08 (0.60, 1.94) | 0.793 |
| **Transgender** |  |  | 0.43 (0.12, 1.55) | 0.196 |
| **Race/Ethnicity** |  |  |  |  |
| **White** |  |  | REF | REF |
| **Black** |  |  | **1.84 (1.15, 2.93)** | **0.011** |
| **Latinx** |  |  | 1.45 (0.82, 2.58) | 0.203 |
| **API** |  |  | 0.72 (0.15, 3.37) | 0.676 |
| **Other** |  |  | 1.48 (0.55, 3.93) | 0.437 |
| **Age** |  |  |  |  |
| **<30** |  |  | REF | REF |
| **30-39** |  |  | 2.09 (0.98, 4.42) | 0.055 |
| **40-49** |  |  | 1.74 (0.78, 3.89) | 0.174 |
| **50-59** |  |  | 1.37 (0.61, 3.11) | 0.445 |
| **60+** |  |  | 1.40 (0.47, 4.15) | 0.547 |
| **Trimorbid** |  |  | 0.90 (0.66, 1.23) | 0.521 |
| **Homeless** |  |  | 0.84 (0.67, 1.04) | 0.112 |

Models adjust for gender (cis-male, cis-female), age by decade, Trimorbidity (e.g having chronic medical, substance use and psychiatric disease based on elixhauser score), homeless (e.g. having reported history of homelessness at any time during the observation period). REF = Reference Level. RR = Relative Risk. OR = Odds Ratio. CI – Confidence Interval . SIP = Shelter in Place. Pre-SIP = RR of having clinical encounter by moth during pre-Shelter in Place observational period. SIP = RR of having clinical encounter at the time of SIP (e.g. March 2020). SIP*Time = interaction term between SIP and time (months). Significant RR indicates a statistically significant different slope from the pre-SIP period. Post-SIP = monthly RR of having clinical encounter following SIP.
